# Music, Media and Mental State: A Comparative Cultural Analysis to Inform Psychiatric Education and Reduce Stigma

**DOI:** 10.1192/bjo.2026.11154

**Published:** 2026-06-30

**Authors:** Pettiann Bhoorasingh

**Affiliations:** King’s College London, London, United Kingdom

## Abstract

**Aims:**

To examine how musicians express mood states through music and how these expressions are interpreted by media narratives, and how cultural analysis can be used as an educational tool to enhance psychiatric understanding of mental state, stigma and patient engagement.

**Methods:**

A qualitative analysis was conducted using a comparative cultural analysis approach. Publicly available materials related to four artists (Amy Winehouse, Kurt Cobain, Stormzy and Florence Welch) were used. These artists were selected to reflect variation across genres, eras, genders and cultural contexts. Materials, such as song lyrics, interviews and media reporting, were thematically analysed using the Braun and Clarke framework. Patterns in emotional expression, representations of mental distress, and media framing were found. Identified themes were mapped to core psychiatric training domains, including mental state variations, stigma and culturally informed care. As no primary clinical data were collected and all materials were in the public domain, ethical approval was not required.

**Results:**

Three consistent themes were identified. First, musicians frequently expressed emotional distress and vulnerability using metaphors, tone and narrative rather than explicit clinical language. This suggests music can provide insight into subjective emotional states and serves as a tool for understanding the meaning and the context of an experience or emotion. Second, media portrayals often simplified or moralised these expressions, particularly in relation to substance abuse, reinforcing stigma and overshadowing the complexity of co-occurring mental health challenges. Third, disparities were observed in how distress was framed, dependent on gender, race and genre, with some artists’ experiences pathologised to a greater extent, whilst others were relatively contextualised or minimised. These findings demonstrate how cultural material can be used in education to show the limitations of symptom-only interpretation and the importance of contextual formulation – including social environment, occupational pressures and media influence. This pedagogical approach aligns with the RCPsych curriculum by enhancing reflective practice and improving cultural competence, pivotal for managing complex presentations in diverse populations.

**Conclusion:**

Music and media analysis offers a potentially useful framework to augment psychiatric training by highlighting how emotional distress is expressed, interpreted and socially constructed. While music cannot be used to diagnose mental illness, it can enhance engagement and facilitate conversations around stigma, identity and help-seeking. Incorporating culturally relevant case analyses through simulated formulation workshops into education may improve recognition of non-verbal expressions of distress, encourage reflective engagement with stigma and media narratives, and promote culturally informed practices.

